# IgM-κ、IgM-λ双克隆型POEMS综合征合并原发性血小板增多症1例

**DOI:** 10.3760/cma.j.cn121090-20241208-00543

**Published:** 2025-08

**Authors:** 娟 王, 凡 吴, 志敏 翟

**Affiliations:** 安徽医科大学第二附属医院血液科，合肥 230601 Department of Hematology, The Second Affiliated Hospital of Anhui Medical University, Hefei 230601, China

患者，75岁男性，因“下肢浮肿伴乏力半月余，发现血细胞异常5 d”于2020年8月1日就诊于我院。病程中双下肢麻木及刺痛感逐渐加重。既往史：肺结核。入院查体：身高170 cm，体重54.5 kg，体温36.7 °C，心率86次/min，呼吸18次/min，血压137/66 mmHg（1mmHg＝0.133 kPa）。阳性体征包括腹部膨隆、脾肿大、移动性浊音阳性、双下肢呈重度凹陷性水肿、总体神经功能限制评分量表（ONLS）下肢评分为4分。完善血常规：WBC 31.45×10^9^/L、HGB 153 g/L、PLT 573×10^9^/L。免疫球蛋白M（IgM）7.54 g/L。促红细胞生成素：正常。胸部CT：双侧胸腔积液、心包积液、脾大。双下肢血管超声未见血栓形成。骨髓涂片：血小板增多骨髓象。骨髓病理：造血组织增生明显活跃；巨核细胞明显增生，弥散或聚集成小簇分布，可见胞体大，核分叶多或鹿角状巨核细胞。诊断骨髓增殖性肿瘤（MPN），首先考虑原发性血小板增多症（ET）。免疫分型：未见异常细胞。染色体：46，XY[20]。JAK2 V617F突变阳性。BCR::ABL融合基因、MYD88 L256突变等均为阴性。入院诊断：ET（IPSET-生存预后评分3分，高危；MIPSS评分6分，高危）。治疗上给予羟基脲降血细胞、阿司匹林抗血小板等。复查血常规：WBC 11.86×10^9^/L、HGB 133 g/L、PLT 246×10^9^/L。但患者的症状和体征并未改善且逐渐出现胸闷。进一步完善血免疫固定电泳：IgMκ及λ在γ区可见单克隆条带。尿免疫固定电泳：κ轻链在γ区可见单克隆条带。血、尿轻链比值正常。肌电图：双下肢周围神经损害（感觉和运动纤维均受累）。胸水提示为渗出液，胸水细菌涂片+培养、脱落细胞学、免疫分型、淋巴瘤基因重排、抗酸染色均未见异常。胸水免疫固定电泳：IgMκ及λ在γ区可见单克隆条带。结核菌素试验：阴性。血清血管内皮生长因子（VEGF）：1 154 pg/ml。甲状腺功能：无异常。性激素：促卵泡生成素15.93 mIU/ml（参考值1.5～12.4 mIU/ml），促黄体生成素9.49 mIU/ml（参考值1.7～8.6 mIU/ml）。心脏增强MRI：左室射血分数71.3％，双侧胸腔及心包积液。腹部超声：脾大、腹盆腔积液（最深处液性暗区49 mm）。PET-CT：骨骼弥漫性18F-氟代脱氧葡萄糖（FDG）代谢增高（SUVmax 7.1），未见骨质破坏。脾大，代谢值未见增高。全身骨平片：未见骨硬化。修正诊断为：POEMS综合征合并ET。治疗上予Rd（来那度胺+地塞米松）方案抗浆细胞，阿司匹林抗血小板。3个月后患者下肢水肿完全消退，影像学显示全身多浆膜腔积液明显减少。6个月后多浆膜腔积液消失，双下肢感觉障碍好转，ONLS评分2分（下肢评分2分）。免疫固定电泳阳性，未达血液学缓解。VEGF降至563 pg/ml。后期患者接受来那度胺及羟基脲治疗，并定期门诊随访，依据血细胞情况动态调整羟基脲用量。截至2024年9月24日，患者一般情况良好，远期疗效进一步随访中。

讨论：POEMS综合征是一种罕见的浆细胞疾病，临床表现多样化，其中超过95％为单克隆轻链，极少数也存在双克隆M蛋白血症。此外，POEMS综合征常伴有继发性血小板增多，但均不存在JAK2 V617F突变。本例患者主要表现为双下肢水肿和乏力、M蛋白双克隆（IgM-κ、IgM-λ）、肌电图为广泛神经元损害、VEGF升高、脾大、多浆膜腔积液、性激素分泌异常，符合双克隆型POEMS综合征的诊断。PLT>450×10^9^/L，骨髓检查符合ET诊断。治疗方面，POEMS综合征主要为积极有效的抗浆细胞治疗。对于不适合移植的患者，存在腹腔大量积液和重度水肿时，建议采用Rd方案诱导治疗。ET需根据危险度分层制定治疗方案，同时预防及治疗血栓并发症，主要为降血细胞、抗血小板或抗凝。结合本病例，我们在前期诊断ET时给予羟基脲降血细胞、阿司匹林抗血小板，症状无改善，随后采取Rd方案抗浆细胞治疗后，其症状在3个月内逐渐好转。提示需根据患者病情制定合理的综合治疗方案。

